# PReS-FINAL-2043: Cardiac involvement in juvenile idiopathic arthritis

**DOI:** 10.1186/1546-0096-11-S2-P56

**Published:** 2013-12-05

**Authors:** E Rossetto, E Benelli, E Mascheroni, E Berton, A Taddio, L Lepore

**Affiliations:** 1 University of Trieste, Trieste, Italy; 2 Institute for Maternal and Child Health-IRCCS, "Burlo Garofolo"-, Trieste, Italy; 3 Institute for Maternal and Child Health-IRCCS, "Burlo Garofolo", Trieste, Italy

## Introduction

Cardiovascular involvement is a relative common complication among adult patients with rheumatic diseases, but, according to our knowledge, few studies have investigated paediatric population.

## Objectives

We investigated the prevalence of cardiovascular involvement in patients with juvenile idiopathic arthritis (JIA).

## Methods

All patients with JIA attending IRCCS Burlo Garofolo in 2012 were enrolled irrespectively from their clinical active disease. Patients enrolled underwent Electrochardiogram (ECG) and complete ecocardiography (two-dimensional, color, continuous, pulsed and tissue Doppler).

## Results

Our sample included 42 patients with juvenile idiopathic arthritis (JIA, mean age at evaluation 13.9 ± 7.9 years, mean disease duration was 6.9 ± 7.6 years, Male/Female ratio:10/32). Among them, 27 (64.3%) were oligoarthritis, 13 (30.9%) polyarthritis, 1 (2.4%) systemic arthritis and 1 (2.4%) psoriasic arthritis. Uveitis were present in 6 patients. Only 5 patients had clinical and laboratory signs of active disease. Among the patients, 36 were receiving a treatment: 22 were in Methotrexate (MTX), 8 were in Etanercept, 4 were in therapy with both MTX and Etanercept, 1 with Adalimumab and 1 with Infliximab. Each patient was matched with a healthy control. We found in the JIA group a slight increased number of valvular alterations: 13 patients with tricuspid insufficiency, 1 with mitral valve insufficiency, 4 with mitral prolaps and 2 with bicuspid aortic valves, while in the control group we found 5 tricuspid insufficiency and 1 aortic insufficiency (p = NS). Two patients had a small pericardial effusion. We found a significant difference between case and controls in systolic and diastolic function both of left (increased Iso Volumetric Contraction Time, IVCT, p < 0.006, and increased Iso Volumetric Realising Time, IVRT, p < 0.001) and right (decreased Tricuspid Annular Plane Systolic Excursion, TAPSE, p < 0.004, and decreased E/A ratio p < 0.004, increased IVRT p < 0.01) ventricles. The correlation between echocardiographic and some clinical parameters (active disease score and the use and duration of drugs) was detected: we found significant correlation only between methotrexate therapy and E/A ratio and IVRT (p < 0.05).

## Conclusion

We found a significant systolic and diastolic abnormalities on both ventricles (decrease in TAPSE and the increase of IVCT and IVRT) in a population affected by JIA. In our opinion it could be attributable to early structural abnormalities (hypertrophy or interstitial fibrosis) caused by prolonged inflammation. Other studies are necessary to understand if these patients might have increased risk of cardiac disease in adulthood, but we think that these patients might benefit from serial cardiac evaluation to highlight early alterations that could require specific therapy.

## Disclosure of interest

None declared.

